# Cardiac Involvement in Human Immunodeficiency Virus Infected Patients: An Observational Cardiac Magnetic Resonance Study

**DOI:** 10.3389/fcvm.2021.756162

**Published:** 2021-11-15

**Authors:** Chengxi Yan, Ruili Li, Xiaojuan Guo, Huan Yu, Wenhuan Li, Wenqiao Li, Meiji Ren, Minglei Yang, Hongjun Li

**Affiliations:** ^1^Department of Radiology, The Second Affiliated Hospital of Xi'an Jiaotong University, Xi'an, China; ^2^Department of Radiology, Beijing Youan Hospital, Capital Medical University, Beijing, China; ^3^Department of Radiology, Beijing Chaoyang Hospital, Capital Medical University, Beijing, China; ^4^Neusoft Research of Intelligent Healthcare Technology, Co. Ltd., Shenyang, China

**Keywords:** HIV, cardiovascular magnetic resonance, cardiac involvement, myocardial inflammation, myocardial fibrosis

## Abstract

**Objectives:** To investigate the subclinical imaging changes in terms of myocardial inflammation and fibrosis and to explore the risk factors associated with myocardial fibrosis by cardiac magnetic resonance (CMR) approach in a Chinese HIV/AIDS cohort.

**Methods:** We evaluated myocardial function (cine), myocardial inflammation (T1, T2), and myocardial fibrosis (through extracellular volume fraction [ECV] and late gadolinium enhancement [LGE]) by a multiparametric CMR scan protocol in a total of 68 participants, including 47 HIV-infected individuals, who were divided into two groups: asymptomatic HIV (HIV+) (n = 30), and acquired immunodeficiency syndrome (AIDS) (*n* = 17), and 21 healthy controls.

**Results:** HIV-infected patients had lower left (55.3 ± 6.5 vs. 63.0 ± 7.9%, *P* < 0.001) and right ventricular systolic function (35.9 ± 15.7 vs. 50.8 ± 9.3%, *P* < 0.001). Radial systolic strain (30.7 ± 9.3 vs. 39.3 ± 9.4%, *P* = 0.001), circumferential systolic strain (−17.5 ± 2.6 vs. −19.4 ± 2.7%, *P* = 0.008), and longitudinal systolic strain (−9.4 ± 5.7 vs. −12.8 ± 3.1%, *P* = 0.012) were also decreased in HIV. Native T1 relaxation time (1,337.2 ± 70.2 vs. 1,249.5 ± 47.0 ms, *P* < 0.001), ECV value (33.5 ± 6.2 vs. 28.5 ± 2.9 ms, *P* = 0.026), and T2 relaxation time (45.2 ± 3.5 vs. 42.0 ± 2.6 ms, *P* = 0.001) were higher in HIV-infected patients compared with controls. Myocardial fibrosis, predominantly in the mid-inferior wall, was detected in 24.4% of the HIV-infected patients. HIV+ had a significantly lower value of ECV [29.1 (26.1, 31.8) vs. 35.2 (31.8, 41.9) %, *P* < 0.001] and frequency of LGE [3/25 (8%) vs. 7/16 (43.8%), *P* = 0.014)] compared with AIDS. AIDS was associated with myocardial fibrosis.

**Conclusions:** HIV-infected patients were associated with changes in myocardial function and higher rates of subclinical myocardial inflammation and fibrosis, which were more abnormal with greater severity of the disease. AIDS was associated with myocardial fibrosis, where the observations supported earlier initiation of antiretroviral therapy in the Chinese HIV/AIDS cohort.

## Introduction

Cardiac abnormalities were believed to be prevalent in HIV infected patients (1, 2). HIV-related cardiomyopathy was reported significantly higher in postmortem studies than in clinical work due to the lack of highly sensitive and specific diagnostic tools (3). Even though the introduction of antiretroviral therapy (ART) has altered the cardiovascular manifestations, HIV-infected patients are still at an increased risk for cardiovascular disease for the high prevalence of traditional cardiovascular risk factors and concurrent metabolic changes induced by ART (4).

Previous studies (5) demonstrated that a major percentage of HIV infected patients have abnormal ECG findings, and echocardiography studies (6, 7) found that patients with asymptomatic HIV infection noted a relatively low frequency of left ventricular dysfunction and other abnormalities when compared with patients in the later stage of the disease. Other imaging modalities (e.g., PET-CT) could help to detect progressing atherosclerosis on blood vessel walls in HIV-infected patients (8) and demonstrated a chronic vascular inflammation resulting from HIV infection. Nevertheless, only a few autopsy studies had been conducted to address myocardial abnormalities in HIV infection (9).

As a technique to assess myocardial structure, function, and also tissue characterization comprehensively, cardiovascular magnetic resonance (CMR) imaging has been widely used in observing myocardial abnormalities. Previous research (10–13) applied CMR or/and magnetic resonance spectroscopy (MRS) to detect cardiac involvement in asymptomatic HIV subjects and found a high burden of cardiac steatosis, decreased myocardial function, and a high rate of myocardial fibrosis in asymptomatic HIV subjects undergoing ART (14). The late gadolinium enhancement (LGE) pattern, as one of the abnormalities revealed by CMR, represented regional fibrosis which might indicate an irreversible myocardial injury and result in cardiac dysfunction and cardiac death (15, 16). Thus, it is crucial to identify risk factors associated with the presence of LGE in HIV-infected patients. In addition, few CMR studies (17) focus on patients in the late stage of HIV disease despite the cardiac complications that occurred more frequently in those patients compared with patients in the early stage. Furthermore, no CMR study to date has assessed the myocardial involvement among the Chinese HIV/AIDS cohort.

Our study aimed to explore the cardiac involvement in Chinese HIV/AIDS patients by CMR while determining the risk factors associated between the HIV-related clinical parameters and myocardial fibrosis.

## Materials and Methods

The institutional ethics committee approved this prospective study, and all participants gave written informed consent prior to CMR.

### Study Participants

Human immunodeficiency virus infected patients were consecutively enrolled in this observational study at Beijing Youan Hospital from June 2019 to July 2020. Inclusion criteria were age ≥18 years and a confirmed HIV diagnosis. Exclusion criteria were history of cardiovascular disease, contraindications for CMR, an estimated glomerular filtration rate of < 90 mL/min/1.73 m^2^, and an impaired liver function (alanine aminotransferase greater than twice the normal upper limit). Clinical histories, physical examinations, and laboratory data were obtained from the enrolled patients, including a detailed review of the HIV disease stage, ART exposure, and cardiovascular disease risk factors. Fasting lipid panels, glucose levels, current plasma CD4^+^ T-cell counts, CD4^+^/CD8^+^ ratios, HIV loads, and hematocrit levels were also acquired. The AIDS stage was defined as the symptomatic stage when the virus becomes highly active and the immune system of the patient weakens, as reported previously (18). The control group consisted of age- and ethnicity-matched (self-defined) subjects (*n* = 21) with no history of HIV infection or cardiovascular disease. CMR studies and subsequent analyses were performed in a blinded manner, with sequential numbering of subjects. Detailed inclusion and exclusion criteria for participants and healthy control participants are illustrated in Supplementary Figure 1.

### CMR Image Acquisition

Cardiac magnetic resonance was performed for patients with 3.0-T systems (MAGNETOM Trio, Siemens Medical Systems, Erlangen, Germany). For cardiac function assessment, steady-state free precession cine images that were electrocardiogram-gated were also performed (short-axis, four-chamber, and two-chamber views). Native and postcontrast T1 mapping was acquired using an ECG-gated single-shot modified Look-Locker inversion recovery (MOLLI) sequence with protocol 5(3)3 and 4(1)3(1)2, respectively, before and 20 min after contrast administration. Late gadolinium enhancement (LGE) imaging based on segmented inversion-recovery gradient-echo sequences was performed 10–15 min after the administration of a single bolus of a 0.2 mmol/kg bodyweight Gadopentetate dimeglumine (Bayer Healthcare, New Jersey). For ECV calculation, blood hematocrit levels were assessed directly prior to the MRI scan.

### CMR Image Analysis

Two readers with 2 (Y.C.X.) and 16 years (G.X.J.) of CMR experience analyzed the data and performed the measurements in consensus using a commercially available software CVI42 (Version 5.11.2 Circle Cardiovascular Imaging, Calgary, Canada).

#### Evaluation of Cardiac Function

To evaluate cardiac function, the endocardial borders of left and right ventricles were automatically detected by the software and manually adjusted, when necessary, in the short-axis cine-steady-state free precession images. Papillary muscles and trabeculae were excluded. Left ventricular end-diastolic volume (LVEDV), right ventricular end-diastolic volume (RVEDV), left ventricular end-systolic volume (LVESV), right ventricular end-systolic volume (RVESV), left ventricular eject fraction (LVEF), right ventricular eject fraction (RVFE), left ventricular cardiac output (LVCO), and right ventricular cardiac output (RVCO) were determined contemporaneously.

#### Feature Tracking

Cardiac magnetic resonance feature tracking (CMR-FT) was performed on short-axis, 4-chamber, and 2-chamber images. Global systolic radial, circumferential, and longitudinal strain values were calculated from peak segmental data. A 16-segment model, according to the American Heart Association model, was applied for segmental strain analysis of the left ventricle (19).

#### T1 and T2 Values

The outline of the endocardium and epicardium were manually contoured on native T1, postcontrast T1, and T2 maps. A 16-segment model was then applied for the acquisition of segmental value. ECV was derived from native T1 and postcontrast T1 of the myocardium as described by the equation in a previous study (20).

#### LGE Images

Images were evaluated qualitatively for the presence or absence, and the volume fraction of LGE was calculated using a threshold of five SDs (21).

### Statistical Analysis

All statistical analyses were performed using SPSS (version 23.0, IBM statistics, Armonk, NY, USA) and GraphPad Prism (Version 8.1, GraphPad Software Inc). Shapiro–Wilk test was used to test the normality of the distribution of the data. Continuous variable with normal distribution or non-normal distribution was expressed as mean ± SD or median (interquartile range). Categorical variables were expressed as counts (percentage). Student's *t-*test (for normal distribution) and unpaired or Mann–Whitney U test (for non-normal distribution) were performed to compare continuous variables between two groups, and χ^2^ test was performed to compare categorical variables. Quantitative variables were transformed into categorical variables according to their normal ranges for univariate logistic regression analysis to identify the association of the presence of LGE and HIV-related parameters. *P*-values of < 0.05 denoted statistically significant.

## Results

### Clinical Characteristics of HIV-Infected Patients and Healthy Controls

Among the 49 HIV-1-infected patients recruited, two patients were excluded because of poor image quality. A total of 47 HIV-infected patients (mean age, 37 ± 9 years, range 23–58 years) were divided into two groups: the asymptomatic HIV (HIV+) group (*n* = 30; 63.83%) and the AIDS group (*n* = 17; 36.2%). Forty-one patients underwent CMR with Gd-contrast material, and six patients refused Gd injections. Twenty-one healthy adults (mean age, 40 ± 11 years, range 20–60) were also evaluated. No significant differences were shown between the HIV-infected participants and healthy controls in age (*P* = 0.332), the occurrence of hypertension rate (*P* = 0.077), heart rate (*P* = 0.251), weight (*P* = 0.409), body mass index (*P* = 0.573), and body surface area (*P* = 0.157). In keeping with previous reports, the ratio of reported infections was four men to one woman by 2018,^1^ and there were more men in the subjects with HIV infection than healthy controls (*P* < 0.001). HIV patients had lower hemoglobin content (135 [126, 143] vs. 164 [136, 163] g/L; *P* = 0.008) compared with healthy controls (as shown in Supplementary Figure 1; Supplementary Table 1).

### Clinical Characteristics of HIV Subgroups

In comparison with HIV+ group, patients in the AIDS group were significantly older (35 [29, 38] vs. 41 [36, 46] years; *P* = 0.010) and had more comorbidity of diabetes mellitus (*P* = 0.035). AIDS group had a longer duration of HIV diagnosis than HIV+ group (6.0 [5.0, 11.5] vs. 3.4 [1.4, 7.3] years, *P* = 0.008). Laboratory results showed a significantly higher current CD4+ (717.2 ± 209.8 vs. 188.2 ± 192.0 cells/mm3; *P* < 0.001), current CD4+/CD8+ ratio (0.68 [0.53, 0.98] vs. 0.14 [0.57, 0.36]; *P* < 0.001), HCT (45.3 [42.8, 47.9] vs. 40.2 [29.9, 43.7] %; *P* < 0.001), and lower glucose (5.1 [4.5, 5.3] vs. 5.6 [4.9, 6.2] mmol/L; *P* = 0.025) in HIV+ group compared with AIDS group. Lower hemoglobin content was shown in the AIDS group compared with the HIV+ group (157 [147, 165] vs. 136 [110, 156] g/dL, *P* = 0.003), (as shown in Supplementary Table 2).

### CMR Characteristics of HIV-Infected Patients and Healthy Controls

Compared with healthy controls, HIV-infected patients had a significantly lower LVEF (55.3 ± 6.5 vs. 63.0 ± 7.9%; P < 0.001), lower RVEF (35.9 ± 15.7 vs. 50.8 ± 9.3%; P < 0.001), lower RVCO (3.5 ± 1.4 vs. 5.0 ± 1.6 l/min; P < 0.001), higher RVESV (83.4 [69.5, 108.2] vs. 61.8 [49.2, 73.3] mL; P < 0.001), and higher RVESVi (83.4 [69.5, 108.2] vs. 61.8 [49.2, 73.3]; P = 0.012) (as shown in Table 1).

**Table 1:** CMR characteristics of HIV patients and healthy controls.

	**HIV Patients**	**Healthy Controls**	***P* Value**
	**(*n* = 47)**	**(*n* = 21)**	
**CMR Parameters**			
**Cardiac Function**			
LVEF (%)	55.3 ± 6.5	63.0 ± 7.9	<0.001^*^
LVEDV (mL)	128.9 ± 36.4	134.9 ± 31.6	0.535
LVESV (mL)	57.0 ± 14.5	49.2 ± 13.4	0.082
LVCO (l/min)	5.5 (4.5, 6.3)	6.0 (4.7, 6.7)	0.259
LVEDVi (mL/m^2^)	64.7 ± 27.8	76.4 ± 16.9	0.086
LVESVi (mL/m^2^)	28.6 ± 12.9	27.7 ± 6.6	0.749
RVEF (%)	35.9 ± 15.7	50.8 ± 9.3	<0.001^*^
RVEDV (mL)	133.2 ± 40.6	133.9 ± 30.8	0.943
RVESV (mL)	83.4 (69.5, 108.2)	61.8 (49.2, 73.3)	<0.001^*^
RVCO (l/min)	3.5 ± 1.4	5.0 ± 1.6	<0.001^*^
RVEDVi (mL/m^2^)	65.3 ± 30.9	75.6 ± 16.5	0.169
RVESVi (mL/m^2^)	45.0 (34.3, 57.7)	34.7 (29.2, 42.8)	0.012^*^
GRS (%)	30.7 ± 9.3	39.3 ± 9.4	0.001^*^
GCS (%)	−17.5 ± 2.6	−19.4 ± 2.7	0.008^*^
GLS (%)	−9.4 ± 5.7	−12.8 ± 3.1	0.012^*^
**Tissue Characterization**			
T2 relaxation time (ms)	45.2 ± 3.5	42.0 ± 2.6	0.001^*^
T1 native (ms)	1337.2 ± 70.2	1249.5 ± 47.0	<0.001^*^
ECV (%)	33.5 ± 6.2	28.5 ± 2.9	0.026^*^
Visual LGE	10/41 (21.95)	0 (0)	0.013^*^
Pericardial effusion	9 (19.1)	4 (19.0)	0.746

*Data are summarized by mean ± SD if they are normal distribution or median (first and third quartiles) if they are abnormal distribution and n (%) for categorical variables. P-values are obtained by using the Student t-test, or Mann–Whitney U test (for non-normal data), X^2^ test, or Fisher exact test. The denominators of patients included in the analysis are provided if they differed from the group's overall numbers. LVEF, left ventricular ejection fraction; LVEDV, left ventricular end-diastolic volume; LVESV, left ventricular end-systolic volume; LVCO, left ventricular cardiac output; LVEDVi, left ventricular end-diastolic volume index; LVESVi, left ventricular end-systolic volume; RVEF, right ventricular ejection fraction; RVEDV, right ventricular end-diastolic volume; RVESV, right ventricular end-systolic volume; RVCO, right ventricular cardiac output; RVEDVi, right ventricular end-diastolic volume index; RVESVi, right ventricular end-systolic volume; GRS, global radial strain; GCS, global radial strain; GLS, global longitudinal strain; ECV, extracellular volume*.

* *Denotes significant values*.

CMR-FT analysis showed that HIV patients had a lower GRS (30.7 ± 9.3 vs. 39.3 ± 9.4%; *P* < 0.001), lower GCS (**–**17.5 ± 2.6 vs. **–**19.4 ± 2.7%; P = 0.008), lower GLS (**–**9.4 ± 5.7 vs. **–**12.8 ± 3.1%; *P* = 0.012) when compared with healthy controls (as shown in Table 1).

**Figure 1:**
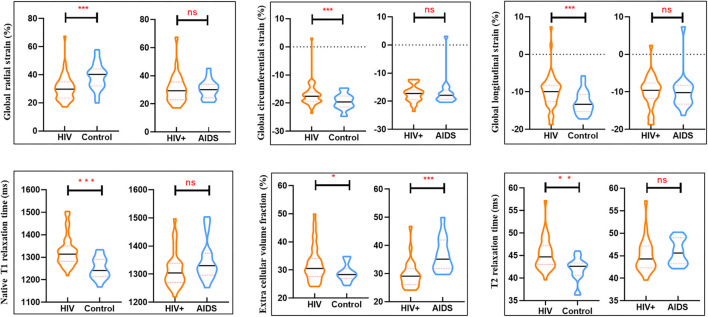
Box plots showed differences in cardiac parameters between groups (HIV group and controls) and within clinically subclassified HIV subgroup (HIV+ and AIDS group). ^*^P < 0.05, ^**^P < 0.01, ^***^P < 0.001; ns no significant difference.

**Figure 2:**
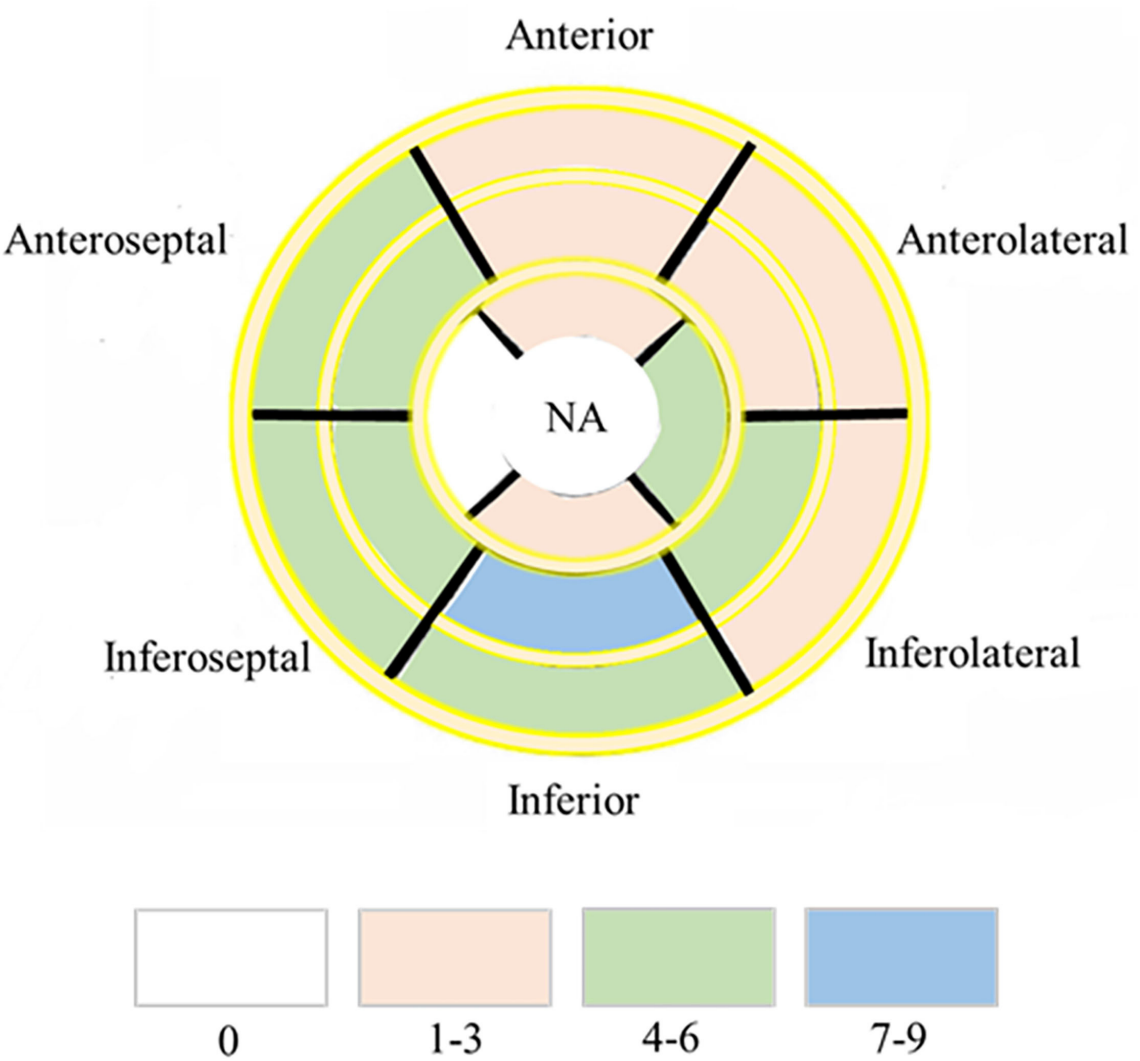
Dominant location and distribution of myocardial fibrosis segments in the AHA 16 segments' model. Number of LGE distributed in AHA 16 segments' model in 41/47 HIV infected patients. NA, not applicable.

Myocardial T1 relaxation times (1,337.2 ± 70.2 vs. 1,249.5 ± 47.0 ms; *P* < 0.001) and ECV (33.5 ± 6.2 vs. 28.5 ± 2.9%; *P* < 0.001) as well as T2 relaxation times (45.2 ± 3.5 vs. 42.0 ± 2.6 ms; P < 0.001) were elevated in participants with HIV patients. The visible sign of myocardial fibrosis was present in a total of 10 of 41 (21.95%) HIV patients, significantly higher than subjects in the control group (*P* = 0.013) (Table 1; Figure 1). Around 7.4% (49 of 656) segments showed LGE and were mainly distributed in the midinferior wall (Figure 2).

### CMR Characteristics of HIV Subgroups

**Figure 3:**
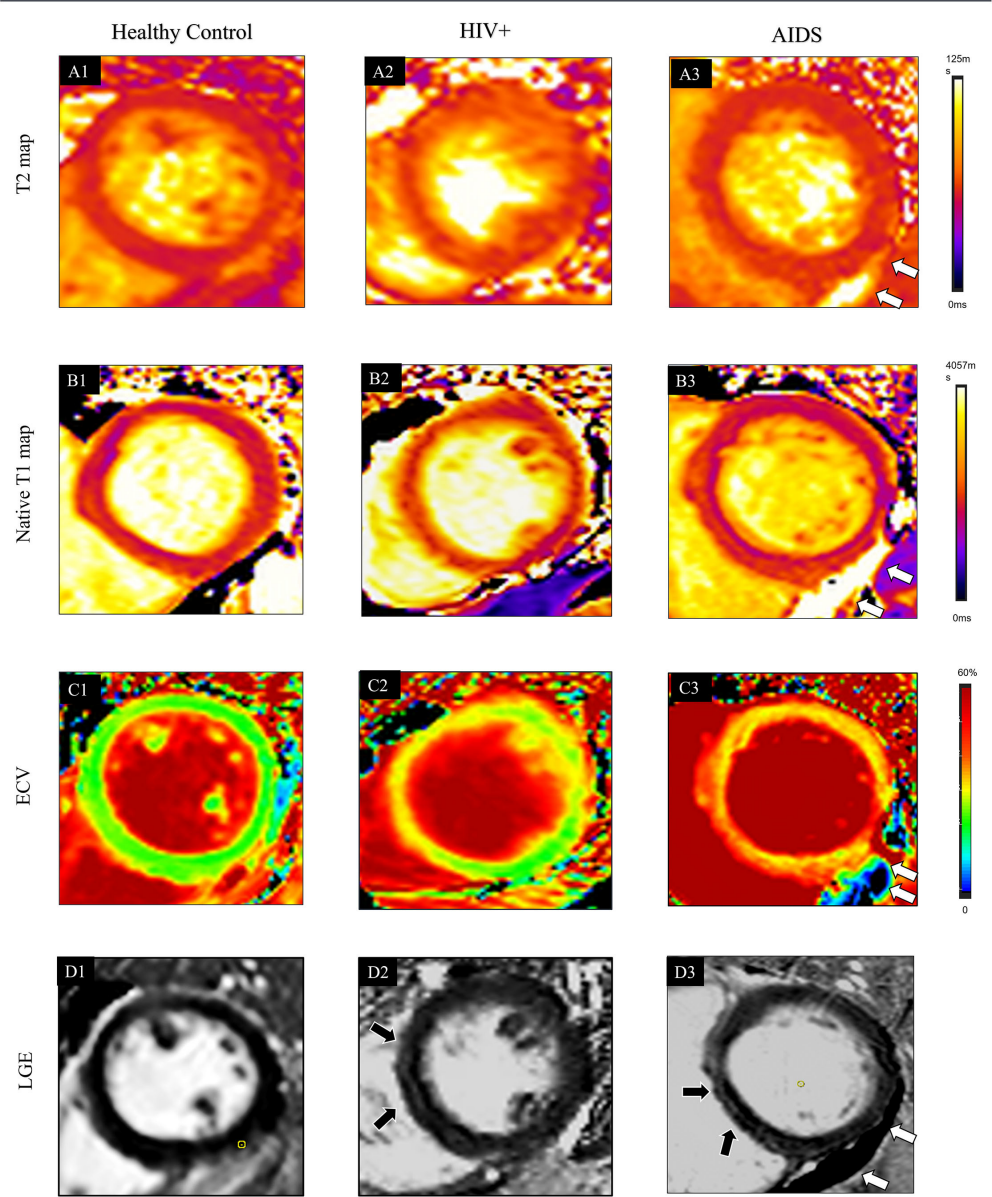
Typical cases showed comprehensive CMR parameters in a healthy control participant (38-year-old man, **A1–D1**), HIV+ patients (47-year-old man, **A2–D2**), and AIDS participant (38-year-old man with the complication of pneumocystis carinii pneumonia; **A3–D3**). Quantitative maps include cardiac T2 relaxation time (T2 map, **A1**), native T1 relaxation time (T1 native map, **B1**), and ECV map (**C1**). Also, LGE images **(D1)** are provided. In the healthy control case, all the quantitative CMR parameters are normal. In HIV+ patients, increased native T1 (1277 ± 209 ms) and ECV (31 ± 6%) are shown in the ventricular septum region of the myocardium on the T1 **(B2)** and ECV maps **(C2)**, which also located in the corresponding region of LGE (**D2**, black arrows). T2 (**A2**, 39 ± 6 ms) values are normal. Global T2 (**A3**, 47 ± 8 ms), T1-native (**B3**, 1314 ± 150 ms), and ECV (**C3**, 33 ± 8%) are further elevated in AIDS patients. LGE **(D3)** pattern is only shown at the ventricular septum area (black arrow). The white arrow indicates pericardial effusion.

There were no significant differences in LVEF, LVEDV, LVESV, LVCO, LVEDVi, LVESVi, RVEF, RVEDV, RVESV, RVCO, RVEDVi, RVESVi between HIV+ and AIDS subjects, nor were there significant differences in GRS, GCS, and GLS. T1-derived ECV measures showed a significantly elevated ECV value in AIDS group compared with HIV+ group (35.2 [31.8, 41.9] vs. 29.1 [26.1, 31.8]%; *P* < 0.001). No differences showed between subgroups in T2 and native T1 relaxation time. Three of the 25 (8%) patients in the HIV+ group showed the presence of LGE, which was significantly lower than those in the AIDS group (seven of 16, 43.8%; *P* = 0.014). Although AIDS patients had a higher volume fraction of LGE compared with HIV+ patients (5.3 [2.1, 10.5] vs. 4.5 [2.8, 7.2]%; P = 0.129), the difference was not significant. There was no difference in the proportion of pericardial effusion between HIV subgroups (*P* = 0.337) (Table 2; Figure 3).

**Table 2:** CMR characteristics of HIV subgroups.

	**HIV + Group**	**AIDS Group**	***P* Value**
	**(*n* = 30)**	**(*n* = 17)**	
**CMR Parameters**			
**Cardiac Function**			
LVEF (%)	55.0 ± 7.0	56.6 ± 6.5	0.499
LVEDV (mL)	123.8 ± 32.3	147.2 ± 36.37	0.095
LVESV (mL)	55.3 ± 16.1	61.6 ± 18.3	0.253
LVCO (l/min)	5.1 ± 1.4	5.8 ± 1.4	0.121
LVEDVi (mL/m^2^)	68.3 ± 17.7	77.4 ± 21.0	0.140
LVESVi (mL/m^2^)	30.5 ± 8.8	33.6 ± 10.8	0.313
RVEF (%)	33.9 ± 7.6	35.5 ± 12.1	0.608
RVEDV (mL)	130.1 ± 35.9	140.9 ± 44.2	0.420
RVESV (mL)	82.2 (72.9, 96.0)	89.7 (63.2, 119.6)	0.468
RVCO (l/min)	3.3 (2.3, 3.9)	3.9 (2.8, 4.7)	0.140
RVEDVi (mL/m^2^)	70.3 (58.3, 79.7)	77.8 (56.4, 89.8)	0.551
RVESVi (mL/m^2^)	45.8 (38.7, 52.1)	52.5 (35.0, 63.3)	0.501
GRS (%)	29.6 (23.0, 35.5)	29.2 (23.7, 33.3)	0.725
GCS (%)	−17.25 (−19.31, −16.0)	−17.8 (−19.3, −16.1)	0.709
GLS (%)	−9.6 (−11.7, −7.6)	−10.0 (−13.3, −8.3)	0.550
**Tissue Characterization**			
T2 relaxation time (ms)	44.5 (42.0, 46.8)	45.5 (43.4, 48.8)	0.189
T1 native (ms)	1309.1 (1270.3, 1346.7)	1330.3 (1296.1, 1374.6)	0.111
ECV (%)	29.1 (26.1, 31.8)	35.2 (31.8, 41.9)	<0.001^*^
Visual LGE	3/25 (8%)	7/16 (43.8%)	0.014^*^
Late gadolinium enhancement, 5sd (%)	4.5 (2.8, 7.2)	5.3 (2.1, 10.5)	0.129
Pericardial effusion (%)	4 (13.3)	5 (29.4)	0.337

*Data are mean ± SD or median (first and third quartiles), the absolute frequency with percentages in parentheses. The denominators of patients included in the analysis are provided if they differed from the group's overall numbers. P-values are obtained by using the Student t-test, Mann–Whitney U test (for non-normal data). X^2^ test, or Fisher exact test*.

* *Denotes significant values, and other abbreviations as in Table 1*.

### Association of Myocardial Fibrosis and HIV Related Clinical Parameters

The results of univariate logistic regression analyses related to cardiac inflammation and fibrosis were shown in Table 3. The clinical factor of AIDS was the risk factor for the presence of LGE (odds ratio 6.300, 95% CI 1.358, 29.235; *p* = 0·019).

**Table 3:** Association of myocardial fibrosis and HIV related clinical parameters.

	**Presence of LGE (*****n*** **= 41)**	
	**Univariable OR (95% CI)**	***P* value**
**Variable**		
Age > 40 y	2.483 (0.273, 22.616)	0.420
Known duration of HIV > 5 years	4.308 (0.950, 19.530)	0.058
ART use	0.906 (0.160, 5.142)	0.921
Current CD4 < 500 cells/mm^3^	1.846 (0.450, 7.573)	0.395
Current CD4+/CD8+ ratio < 1.4%	1.091 (0.108, 11.012)	0.941
Plasma HIV RNA > 75 copies/ml	1.554 (0.326, 7.413)	0.581
AIDS	6.300 (1.358, 29.235)	0.019^*^

*OR, odds ratio; ART, antiretroviral therapy*.

* *Denotes significant values. Other abbreviations as in Table 1*.

## Discussion

To the best of our knowledge, it was the first study to provide an extensive assessment of the cardiac structure, function, and myocardial tissue characterization using a comprehensive CMR approach in the Chinese HIV/AIDS cohort. The novel key finding of this observational, prospective study was the high burden of cardiac disease with signs of focal and diffuse myocardial edema and fibrosis, as well as a high incidence of pericardial effusion, indicating the subclinical myocardial inflammation in HIV-infected patients. ECV value and LGE presence were higher depending on the stage of HIV disease, revealing a link between the clinical grade of HIV disease and cardiac fibrosis. Besides, AIDS was a risk factor of myocardial fibrosis, further supporting the proposal that all patients with HIV should be commenced on ART to control the development of the disease.

T2 mapping was widely used to detect edema and inflammation in myocardial disease (22); however, it was poorly investigated in the context of HIV-related cardiomyopathy to date. Our findings of elevated T2 relaxation time in HIV-infected patients, when compared with healthy controls, indicated the presence of edema which was also evidence of inflammation (23). A slight increase in T2 relaxation time was shown in the AIDS group compared with HIV+, but the difference was not significant. Gutberlet et al. (24) pointed out that T2-weighted imaging had lower sensitivities as well as specificities in patients with suspected chronic myocarditis when compared with acute myocarditis. Abdel-Aty et al. (25) also found that T2-weighted CMR was sensitive not only to edema-related signal changes but also to a variety of pathological phenomena that occurred during infarct healing so that it could not differentiate acute from chronic myocardial infarction. T2 relaxation times are negatively correlated to collagen content in muscle tissue (26). A higher ratio of fibrosis tissue in chronic myocardial injury may result in the apparent “normalization” of T2 value (27) as observed in our study.

Another CMR parameter for quantification of myocardial inflammatory is native T1 relaxation time (28, 29). Higher native T1 values, as shown in our study, probably represented a combination of patchy myocardial edema, fibrosis, and inflammation. LGE existed in 10 of 41 (24.2%) HIV patients, and participants in the AIDS stage (43.8%) had a higher occurrence rate of LGE than HIV+ (8%). LGE, as a part of the healing response to subclinical myocarditis, might represent irreversible myocardial injury as previously described for the non-infected population (15). The presence of LGE predicted a worse outcome than did the absence of LGE (30). In accordance with the LGE pattern, a significant difference of ECV values which were known to represent an indirect measure of diffuse interstitial myocardial fibrosis (31) was shown between patients and controls and also HIV subgroups. Myocarditis was reported frequently on biopsy in the majority of HIV patients during the pre-ART era (32), whereas in the ART era, subclinical myocarditis has not been reported extensively. Even though most of the HIV participants in our study (37 of 47, 78.7%) received ART, myocardial inflammation still seems to prevail. We postulated immune restoration that is mediated by ART was incomplete even in long-term virologically suppressed patients, and the immune dysfunction could translate to a higher risk for developing chronic diseases (33). Besides, ART side effects should also be recognized as a possible confounding factor of systemic inflammation (34). Effusion was also shown in our study with the occurrence rate of 19.1%, and the ratio was even higher in AIDS group (29.4%) compared with HIV+ group (13.3%). The higher sensitivity of CMR has enabled the demonstration of small pericardial effusions which likely support the presence of a higher degree inflammatory state in the AIDS group.

A striking tendency was found toward a reduced ejection fraction of left ventricular in HIV-positive subjects and it further decreased in the AIDS group; besides, GRS, GCS, and GLS values were all decreased in HIV patients, which were in line with previous echocardiography and CMR studies (10, 11, 13, 35). HIV-associated cardiomyopathy often occurred as reduced LVEF or dilated LV, and studies with a higher proportion of AIDS reported a higher prevalence of left ventricular dysfunction (36). The mechanisms of cardiac dysfunction remain unclear. There are several pathogens: HIV-infected patients are more prone to have subclinical coronary atherosclerosis due to HIV infection and antiretroviral therapy (37). As reported by a previous study that long-term exposure to antiretroviral medications was prone to substantial metabolic risk, with about 50% of patients having dyslipidemias and 1/3 had accompanying impaired glucose tolerance (38). We also found a higher glucose content in AIDS patients compared with HIV+ patients (5.6 vs. 5.1 mmol/L), which may further lead to cardiovascular risk (38). Subclinical myocardial inflammation as revealed by CMR further predisposed to left myocardial dysfunction. Besides, HIV therapy may further compound cardiovascular function. Nucleoside analog reverse-transcriptase inhibitors, such as zidovudine and stavudine, may have marked negative effects on myocardial structure through mitochondrial toxicity inhibition of the mtDNA replicate and DNA pol-γ (39).

**Figure 4:**
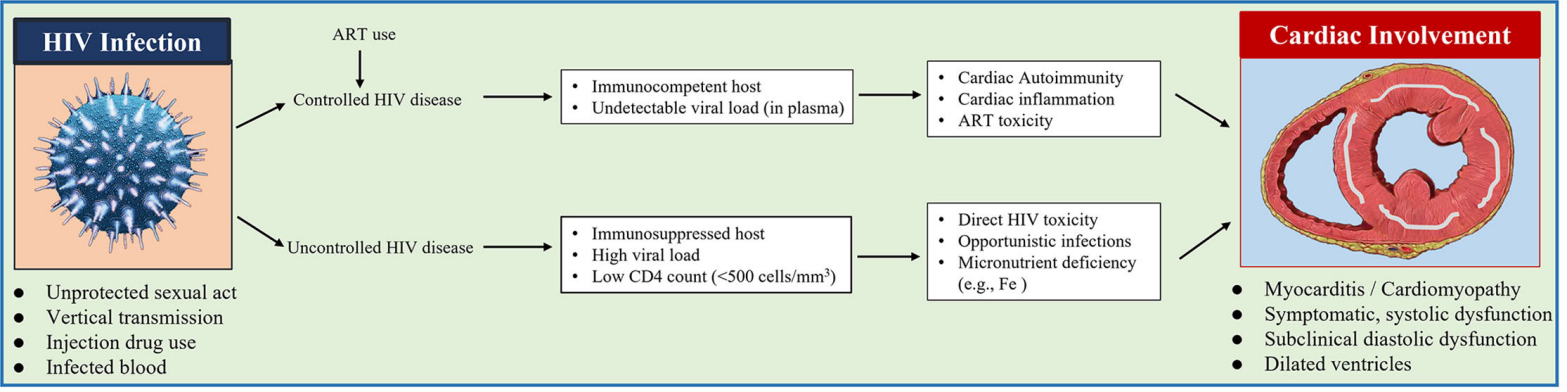
Diagram shows the proposed pathogenic mechanism of cardiac involvement in patients with HIV disease.

Cine-MRI data also confirmed RV anatomic alterations and also RV function impairment in HIV subjects. Casalino et al. (40) demonstrated that when using echocardiography, AIDS patients had a significantly increased RVESV and deceased RVEF compared with healthy controls. The thinner-walled RV tends to manifest myocardial abnormalities and structural and functional modifications (41). Pulmonary hemodynamic disturbances as well as fluid loss caused by diarrhea, which was common during HIV infection and was believed to explain RV modifications in AIDS (42). The diagram shows the proposed pathogenic mechanism of cardiac involvement in patients with HIV disease (Figure 4).

There were still several limitations because of the observational and explorative study design. The exact causality or pathogenesis for our findings cannot be determined since there is no endomyocardial biopsy. Yet, several studies have validated these applied CMR parameters against histologic analysis in the detection of myocardial fibrosis and inflammation (43). Second, in this Chinese HIV cohort, 45 of 47 (95.74%) patients were men; therefore, these results are likely not generalizable to women. However, this may reflect the gender distribution of the AIDS epidemic in China (44). Further study with a larger sample size of women was needed to confirm these findings before the study results may be generalized. Third, patients in the AIDS stage were limited in numbers. Therefore, research with a larger sample size of AIDS would be recommended in the future. Finally, ART use might be confounders. However, in accordance with previous studies, cardiac involvement in HIV patients was independent of the virological suppression and was observed in participants on ART as well as in those naïve to ART (11).

## Conclusions

In conclusion, the comprehensive CMR approach showed a high burden of myocardial structural and functional abnormalities as well as chronic inflammation, which intensify with the severity of HIV disease; besides, AIDS was associated with myocardial fibrosis. Our findings prompt the need for closer cardiologic check-ups (e.g., for cardiac medication adjustment or antiinflammatory therapy), especially in patients of late-stage HIV disease or if imaging-based inflammation markers are particularly high.

## Data Availability Statement

The original contributions presented in the study are included in the article/Supplementary Material, further inquiries can be directed to the corresponding author/s.

## Ethics Statement

The studies involving human participants were reviewed and approved by the Institutional Ethics Committee of Youan approved this prospective study. The patients/participants provided their written informed consent to participate in this study. Written informed consent was obtained from the individual(s) for the publication of any potentially identifiable images or data included in this article.

## Author Contributions

CY: conceptualization, formal analysis, investigation, data curation, writing—original draft, and writing—review and editing. RL: formal analysis, investigation, data curation, writing—original draft, and writing—review and editing. XG: formal analysis, investigation, and resource. HY and WenhL: review and editing. WenqL and MR: data curation. MY: software and writing—review and editing. HL: conceptualization, writing—review and editing, resource, and supervision. All authors contributed to the article and approved the submitted version.

## Funding

This work was supported by the National Natural Science Foundation of China [Grant No. 81771806 and 61936013]; Beijing Natural Science Foundation [7212051]; Beijing Excellent Talent Plan [Grant No. 2018000021469G290].

## Conflict of Interest

Author MY was employed by Neusoft Research of Intelligent Healthcare Technology, Co. Ltd. The remaining authors declare that the research was conducted in the absence of any commercial or financial relationships that could be construed as a potential conflict of interest.

## Publisher's Note

All claims expressed in this article are solely those of the authors and do not necessarily represent those of their affiliated organizations, or those of the publisher, the editors and the reviewers. Any product that may be evaluated in this article, or claim that may be made by its manufacturer, is not guaranteed or endorsed by the publisher.

## Footnotes

^1^https://www.cdc.gov/hiv/group/gender/men/index.html.
