# Supplementary material for: Cardiac Involvement in Human Immunodeficiency Virus Infected Patients: An Observational Cardiac Magnetic Resonance Study
Source: Front Cardiovasc Med. 2021 Nov 15;8:756162. doi: 10.3389/fcvm.2021.756162 (PMC8634394; doi:10.3389/fcvm.2021.756162)

**Supplementary Table 1: Clinical Characteristics of HIV subjects and Healthy Control Participants**

|  | HIV Participants  (n=47) | Healthy Control Participants (n=21) | *P* Value |
| --- | --- | --- | --- |
| Variable |  |  |  |
| Demographic and blood pressure |  |  |  |
| Age (y) | 37 (30,42) | 37 (33, 49) | 0.332 |
| Men | 45 (95.7) | 12 (57.1) | <0.001* |
| Hypertension | 9 (19.1) | 0 (0) | 0.077 |
| Heart rate (bpm) | 69±8 | 68±9 | 0.251 |
| Hemoglobin (g/dl) | 135 (126, 143) | 164 (136, 163) | 0.008* |
| Anthropometric measurements |  |  |  |
| Weight (kg) | 70 (64, 78) | 65 (60, 80) | 0.409 |
| Body mass index (kg/m²) | 23.0 (21.0, 24.5) | 23.1 (20.8, 26.8) | 0.573 |
| Body surface area (m²) | 1.8 (1.7, 1.9) | 1.7 (1.7, 1.9) | 0.157 |

Data are summerized by mean±SD if they were abnormal distribution or median and interquartile range if they were abnormal distribution and n (%) for categorical variables. *P* values were obtained by using Student t test, or Mann–Whitney U test (for non-normal data), X² test or fisher exact test.

**Supplementary Table 2:Clinical Characteristics of HIV Subgroups**

|  | HIV (n=30) | AIDS (n=17) | *P* Value |
| --- | --- | --- | --- |
| Variable |  |  |  |
| Demographics and clinical characteristics |  |  |  |
| Age (y) | 35 (29, 38) | 41 (36, 46) | 0.010* |
| Men | 28 (96.7) | 17 (100) | 0.553 |
| Hypertension | 6 (20.0) | 3 (17.6) | 0.862 |
| Heart rate (bpm) | 68±3 | 67±10 | 0.183 |
| DM | 2 (6.7) | 5 (29.4) | 0.035* |
| Smoking history | 11 (36.7) | 7 (63.4) | 0.165 |
| HIV acquisition risk |  |  |  |
| IVDU | 3 (10.0) | 3 (17.6) | 0.450 |
| MSM | 23 (76.7) | 4 (41.4) | 0.014* |
| Heterosexual | 1 (3.3) | 4 (23.5) | 0.030* |
| Blood transfusion recipients | 3 (10.0) | 3 (17.6) | 0.450 |
| Known duration of HIV diagnosis (y) | 3.4 (1.4, 7.3) | 6.0 (5.0, 11.5) | 0.008* |
| ART use |  |  |  |
| Current | 28 (93.3) | 9 (76.5) | 0.090 |
| Duration (y) | 2.5 (1.5, 5.1) | 1.5 (0.3, 6.3) | 0.424 |
| Anthropometric measurements |  |  |  |
| Weight (kg) | 70.0 (64.5, 80.0) | 70.0 (63.5, 76.5) | 0.665 |
| Body mass index (kg/m²) | 22.9 (21.0, 25.4) | 23.3 (20.0, 24.4) | 0.432 |
| Body surface area (m²) | 1.8 (1.7, 1.9) | 1.8 (1.7, 1.9) | 0.799 |
| Laboratory results |  |  |  |
| Current CD4 (cells/mm³) | 717.2±209.8 | 188.2±92.0 | <0.001* |
| Current CD4+/CD8+ ratio (%) | 0.68 (0.53, 0.98) | 0.14 (0.36, 0.57) | <0.001* |
| Plasma HIV RNA, copies/ml |  |  |  |
| <75 | 26 (86.7) | 9 (52.9) | <0.001* |
| 75—9,999 | 3 (10.0) | 3 (17.6) | .. |
| >10,000 | 1 (3.3) | 5 (29.4) | .. |
| HCT (%) | 45.3 (42.8, 47.9) | 40.2 (29.9, 43.7) | 0.001* |
| Hemoglobin (g/dL) | 157 (147, 165) | 136 (110, 156) | 0.003* |
| Creatine (umol/L) | 69.6±11.2 | 66.4±17.2 | 0.147 |
| Glucose (mmol/L） | 5.1 (4.5, 5.3) | 5.6 (4.9, 6.2) | 0.025* |
| Triglyceride level (mg/dL） | 1.9 (1.2, 3.7) | 1.3 (0.8, 2.5) | 0.084 |
| Cholesterol level (mmol/L) |  |  |  |
| Total | 5.0±1.0 | 4.0±1.1 | 0.899 |
| HDL | 0.96 (0.81, 1.11) | 0.83 (0.62, 1.00) | 0.065 |
| LDL | 2.9±1.0 | 2.2±0.9 | 0.329 |

Data are mean±SD or median (first and third quartiles), absolute frequency with percentages in parentheses. The denominators of patients who were included in the analysis are provided if they differed from the overall numbers in the group. *P* values were obtained by using one-way X² test, fisher exact test or Mann–Whitney U test (for non-normal data). *IVDU* intravenous drug users, *MSM* men who have sex with men, *ART* antiretroviral therapy, *HCT* hematocrit, *HDL* high-density lipoprotein, *LDL* low-density lipoprotein. *Denotes significant values.

**Supplemental Figure 1: Flowchart of Study Design**


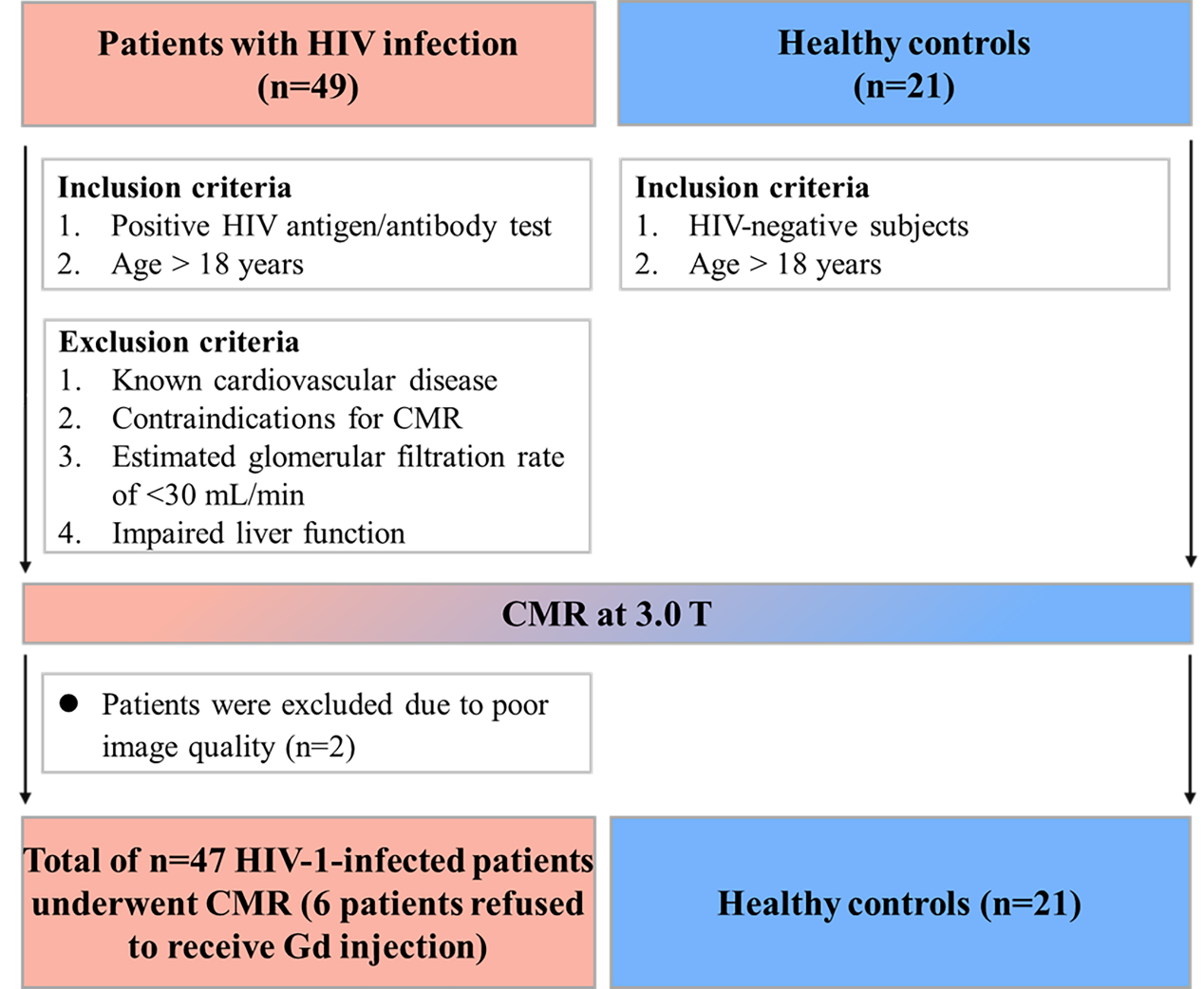

Supplement: Supplementary file 1 [file Data_Sheet_1.docx]
