# Supplementary figures and images for: Cardiac Involvement in Human Immunodeficiency Virus Infected Patients: An Observational Cardiac Magnetic Resonance Study
Source: Front Cardiovasc Med. 2021 Nov 15;8:756162. doi: 10.3389/fcvm.2021.756162 (PMC8634394; doi:10.3389/fcvm.2021.756162)

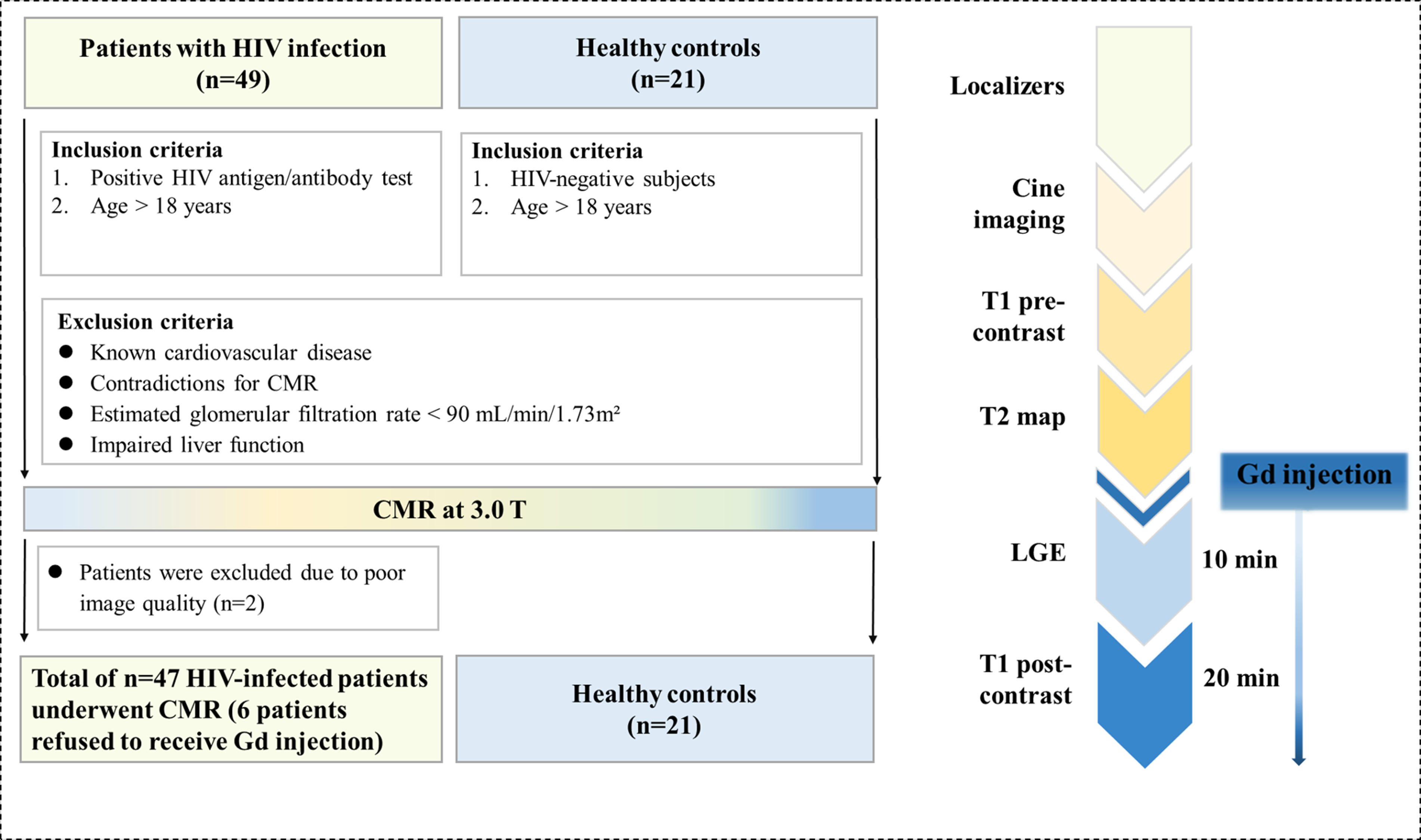

Supplement: Supplementary file 2 [file Image_1.TIFF]
